# Benign Subcutaneous Emphysema of the Lower Limb Following a Trivial Injury: A Case Report

**DOI:** 10.7759/cureus.111040

**Published:** 2026-06-17

**Authors:** Ravi K Pathak

**Affiliations:** 1 Department of Orthopaedics, NPI Narayani Samudayik Hospital, Bharatpur, NPL

**Keywords:** benign subcutaneous emphysema, conservative management, gas gangrene mimic, lower limb trauma, puncture wound, soft tissue swelling

## Abstract

Subcutaneous emphysema involves air entrapment beneath the subcutaneous tissue and fascial planes. It can range from a benign condition to a life-threatening emergency. Benign subcutaneous emphysema is typically confined to the subcutaneous tissues, lacks systemic features, and is non-progressive. We report a case of benign subcutaneous emphysema following a trivial trauma to the left leg of a 16-year-old male. Examination revealed a localized puncture wound and palpable crepitus. Following debridement of the puncture wound, the patient was managed conservatively with a below-knee posterior slab and antibiotics. Complete resolution of symptoms was achieved after two weeks. Subcutaneous emphysema in the lower limb is a rare, benign condition that must be carefully distinguished from severe, life-threatening necrotizing soft-tissue infections such as gas gangrene. While infectious etiologies demand immediate, aggressive surgical intervention, benign variants can be safely managed conservatively if the patient's systemic condition remains stable. Temporary joint immobilization prevents the progression of the "ball-valve" mechanism, leading to complete resolution within one to three weeks. Proper clinical evaluation through a meticulous history, physical examination, and basic radiological investigations helps differentiate benign subcutaneous emphysema from lethal surgical emergencies. Early identification guides appropriate conservative management, preventing unnecessary surgical intervention and extensive, prolonged antibiotic treatment.

## Introduction

Subcutaneous emphysema is characterized by the entrapment of air underneath the subcutaneous tissue and fascial planes, resulting in localized soft-tissue swelling. The most frequent etiologies include surgical procedures, trauma, and gas-producing infections. The diagnosis of subcutaneous emphysema is predominantly clinical, with palpable crepitus over the affected swelling serving as a diagnostic hallmark [1].

Subcutaneous emphysema more commonly occurs in the upper extremity. Its isolation in the lower limb following non-operative, trivial injuries remains exceptionally rare, representing less than 5% of reported non-surgical cases. When it occurs in the lower extremity, it can lead to severe diagnostic dilemmas. The primary clinical complication is misdiagnosis, which can result in either unnecessary, aggressive surgical debridement for a benign condition, or conversely, a catastrophic delay in treating lethal, rapidly progressive conditions like necrotizing fasciitis or gas gangrene [2,3].

We report this case to demonstrate that minor trauma can induce a benign, purely mechanical variant of lower limb emphysema. Awareness of this distinct entity ensures the implementation of safe, conservative management, sparing patients from unnecessary surgery and inappropriate broad-spectrum antibiotic use.

## Case presentation

A 16-year-old boy presented to our emergency department following a fall injury while playing football. He complained of pain and swelling localized around his left ankle and distal leg. He noted that he sustained a minor puncture wound over the anterior aspect of his ankle from a wooden stick during the fall. There was no history of trauma to any other anatomical sites.

**Figure 1:**
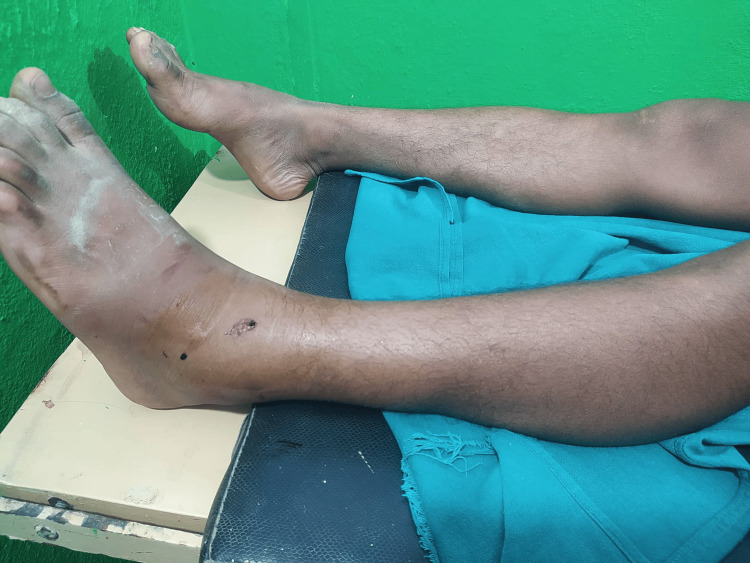
Puncture wound over the anterior aspect of the left ankle with localized swelling of the distal leg

On physical examination, the patient was fully oriented, cooperative, and hemodynamically stable. His vital signs were well within normal physiological limits: axillary temperature was 37.1 °C (98.8°F), blood pressure was 114/72 mmHg, heart rate was 74 beats per minute, and respiratory rate was 16 breaths per minute. Local examination revealed a 5 mm puncture wound over the anterior aspect of the left ankle (Figure 1).

Crucially, the surrounding skin was cool to the touch, with no active bleeding, purulent discharge, foul odor, or advancing erythema. Widespread soft-tissue swelling and prominent crepitus were palpable across the distal leg and around the ankle joint, but these findings were confined strictly to the superficial layers without underlying fluctuance, structural induration, or disproportionate exquisite tenderness. The passive and active ranges of motion of both the ankle and knee joints were full and painless.

Investigations

**Figure 2:**
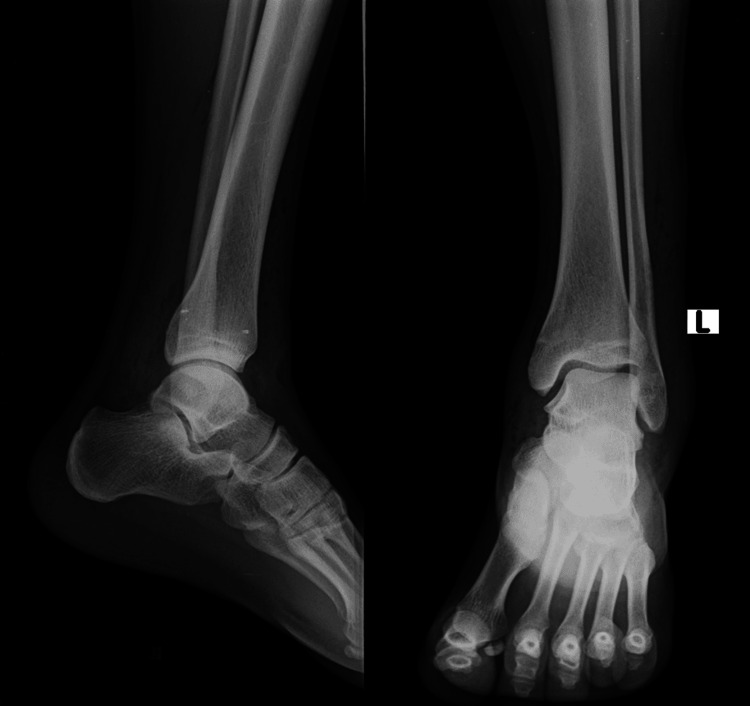
Plain radiographs (anteroposterior and lateral views) demonstrating the presence of air pockets localized within the subcutaneous tissues surrounding the ankle and extending into the distal leg

Initial laboratory investigations did not reveal any significant systemic inflammatory process. The white blood cell (WBC) count was 6500/mm^3^, and the C-reactive protein (CRP) was non-reactive. Plain radiographs (anteroposterior and lateral views) of the left ankle and leg demonstrated the distinct presence of air pockets localized within the subcutaneous tissues surrounding the ankle and extending into the distal leg (Figure 2).

Treatment and outcome

The patient was admitted for observation and started on empirical prophylactic IV antibiotics consisting of IV flucloxacillin 500 mg every 6 hours and IV clindamycin 300 mg every 8 hours to prevent secondary bacterial infection. Thorough local debridement of the puncture wound was performed under aseptic conditions, followed by the application of a sterile dressing. Because the injury occurred outdoors on a football field, the patient's immunization status was assessed, and a 0.5 mL intramuscular injection of tetanus toxoid was administered prophylactically in the emergency department. To minimize limb movement, a below-knee posterior plaster slab was applied.

During 48 hours of inpatient observation, the patient remained afebrile, and vitals remained stable. Repeat laboratory parameters showed no signs of escalating inflammation, and the localized swelling did not progress. He was subsequently discharged on oral antibiotics consisting of oral flucloxacillin 500 mg every 6 hours and oral clindamycin 300 mg every 8 hours to complete a total 7-day course of antimicrobial therapy. The patient was reviewed weekly in the outpatient department. At the second-week follow-up, the below-knee posterior slab was removed after confirming the puncture wound had healed completely. There was total resolution of the clinical crepitus and subcutaneous emphysema.

## Discussion

Subcutaneous emphysema isolated to the lower limb following non-operative trauma is an extremely rare clinical entity [2]. Because of the clinical presentation, it mimics lethal soft-tissue emergencies, making it vital to distinguish it from gas gangrene and necrotizing fasciitis. Patients suffering from necrotizing soft-tissue infections typically appear highly toxic and septic, exhibiting severe localized inflammatory signs (discoloration, hemorrhagic bullae, disproportionate pain) and marked leukocytosis or elevated inflammatory markers [3,4]. Radiologically, malignant emphysema shows gas dissecting along deep fascial lines or within muscle planes, requiring emergent surgical debridement and fasciotomy [4,5].

In contrast, benign subcutaneous emphysema follows a mechanical break in the skin layer. It presents with a stable general clinical condition, localized superficial crepitus, normal laboratory findings, and gas strictly limited to the subcutaneous layer on plain radiographs [2]. The underlying mechanism involves a mechanical disruption where the broken skin acts as a temporary one-way valve, sucking air into tissues during movement. Physical activity or limb movement increases the efficiency of this "ball-valve" mechanism, accelerating the accumulation of subcutaneous air [2,5]. In our patient's specific scenario, the repetitive, high-demand ankle plantarflexion and dorsiflexion while running during his football match served as an active mechanical pump, forcefully driving ambient atmospheric air through the 5 mm puncture wound into the loose subcutaneous fascial planes.

To securely differentiate this benign presentation from lethal surgical infections, a brief hospital admission for close observation (24 to 48 hours) is highly recommended. Serial clinical examinations during this window allow the care team to visually confirm that the crepitus is non-progressive and that the patient remains entirely afebrile and systemically well.

Reported non-infectious causes include blast injuries, air-gun trauma, dental procedures, and wound irrigation with hydrogen peroxide. Most authors agree that benign variants are successfully managed via conservative protocols. Depending on wound contamination, management includes local wound toilet or minor debridement [6]. Prophylactic antibiotic coverage should be initiated to prevent secondary bacterial infection. Our use of flucloxacillin and clindamycin provided ideal empirical cover against common Gram-positive skin pathogens while capitalizing on clindamycin's excellent anaerobic coverage. As recommended by Crampton, immediate immobilization of the affected limb is crucial because it halts the mechanical ball-valve tracking, preventing further migration of trapped air [7]. Complete resolution is typically achieved within one to three weeks as the body gradually reabsorbs the trapped gas.

## Conclusions

Not all presentations of subcutaneous emphysema indicate necrotizing fasciitis or gas gangrene. This case demonstrates that a benign, mechanical variant can present in the lower limb following trivial sports trauma. The key clinical takeaway is that when a patient presents with localized crepitus but remains hemodynamically stable and afebrile, and shows normal inflammatory markers with gas confined strictly to the superficial tissue on radiographs, aggressive surgical intervention is entirely unwarranted. Clinicians should confidently manage these cases via local wound care, prophylactic antibiotics, and temporary joint immobilization, coupled with a brief 48-hour observational window to safely rule out necrotizing soft-tissue infections.
